# Supplementary material for: Effects of marker type and filtering criteria on QST-FST comparisons
Source: R Soc Open Sci. 2019 Nov 6;6(11):190666. doi: 10.1098/rsos.190666 (PMC6894560; doi:10.1098/rsos.190666)
Supplement: Supplementary Tables & Figures [file rsos190666supp1.pdf]

Supporting information for manuscript "Effects of marker type and filtering criteria on Q_{ST}-F_{ST} comparisons"

Zitong Li[1†], Ari Löytynoja[2†], Antoine Fraimout[1]* and Juha Merilä[1]

[1]*Ecological Genetics Research Unit, Department of Biosciences, FI-00014 University of Helsinki, Finland*.
[2]*Institute of Biotechnology, FI-00014 University of Helsinki, Finland*

[†]These authors contributed equally to this work

*Corresponding author: *Ecological Genetics Research Unit, Department of Biosciences, FI-00014 University of Helsinki, Finland*. E-mail: antoine.fraimout@helsinki.fi

Table of contents

**Fig. S1. Estimation of recombination rate for microsatellite loci.**	**Page 3**
**Fig. S2. Distribution of per locus pairwise F_{ST} for HEL-LEV comparison divided by recombination rate and allelic richness of microsatellite loci.**	**Page 4**
**Figure S3. Schematic representation of the demographic scenario used to simulate *P. pungitius* genomic datasets.**	**Page 5**
**Fig. S4. Variation at the microsatellite loci across the four nine-spined stickleback populations.**	**Page 6**
**Figure S5. H statistic from Driftsel using ascertained and unascertained markers.**	**Page 7**
**Figure S6. Results of Driftsel and QstFstComp analyses based on simulated data.**	**Page 8**
**Table S1. S and H statistics from driftsel analysis using three different SNP datasets and unascertained markers.**	**Page 9**
**Table S2. S and H statistics from driftsel analysis using *in-silico* genotyped microsatellite markers and unascertained markers.**	**Page 10**
**Table S3. F_{ST}-Q_{ST} differences and associated *p*-values from QstFstComp analysis using three different SNP datasets.**	**Page 11**
**Table S4. Results from QstFstComp analysis based on microsatellite markers**	**Page 12**
**Table S5. S and H statistics from Driftsel analysis using three different SNP datasets and ascertained markers.**	**Page 13**
**Table S6. S and H statistics from driftsel analysis using *in-silico* genotyped**	**Page 14**

**microsatellite markers and ascertained markers.**	
**Table S7. F_{ST}-Q_{ST} differences and associated *p*-values from QstFstComp analysis using three different SNP datasets after deleting ascertained markers.**	**Page 15**
**Table S8. Toy illustration of the differences in LD structures in marine and pond populations.**	**Page 16**
**Table S9. Number of detected signals of selection among 10 simulation replicates using Driftsel and QstFstComp.**	**Page 17**

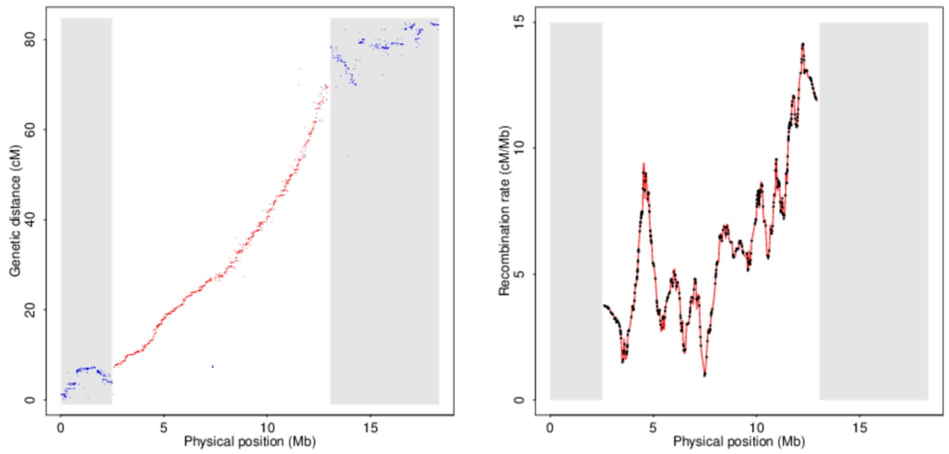

Fig S1. Estimation of recombination rate for microsatellite loci. a) Based on a Marey map, here for LG3, inconsistent regions (gray shading) and markers (in blue) were removed. b) The remaining loci were used to estimate the local recombination rate across the sites (red line) and for individual loci, here microsatellites (black circles).

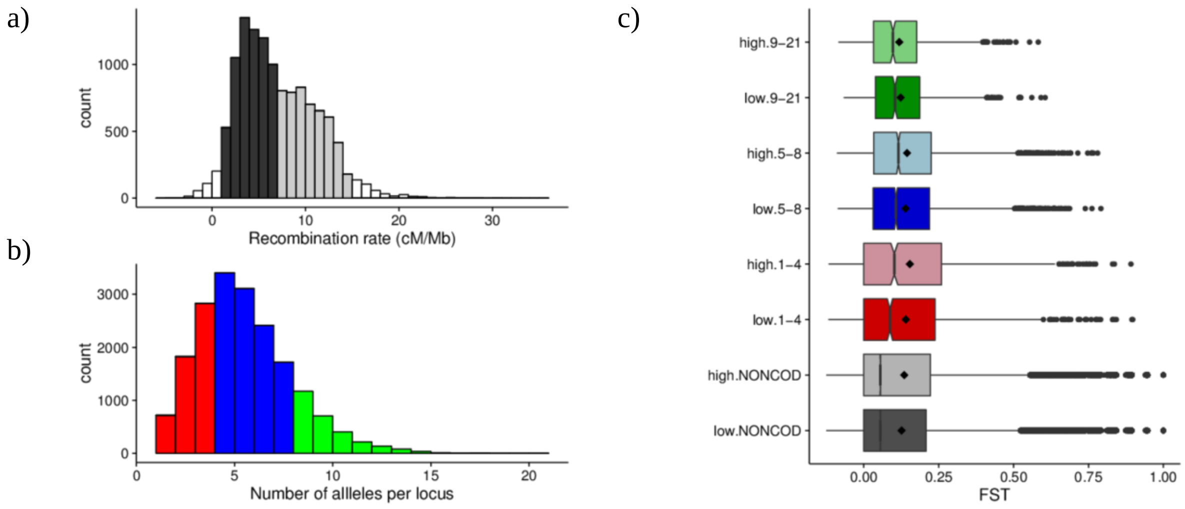

Fig S2. Distribution of per locus pairwise F_{ST} for HEL-LEV comparison divided by recombination rate and allelic richness of microsatellite loci. Distribution of a) recombination rate and b) allele number per locus for 12,207 microsatellite loci. The loci are split by their recombination rate ("low" in dark gray, "high" in light gray; loci marked in white were discarded), and number of alleles (1-4 in red, 5-8 in blue, 9-21 in green). c) Distribution of F_{ST} for the two categories of SNP dataset NONCOD and the six categories of microsatellite loci.

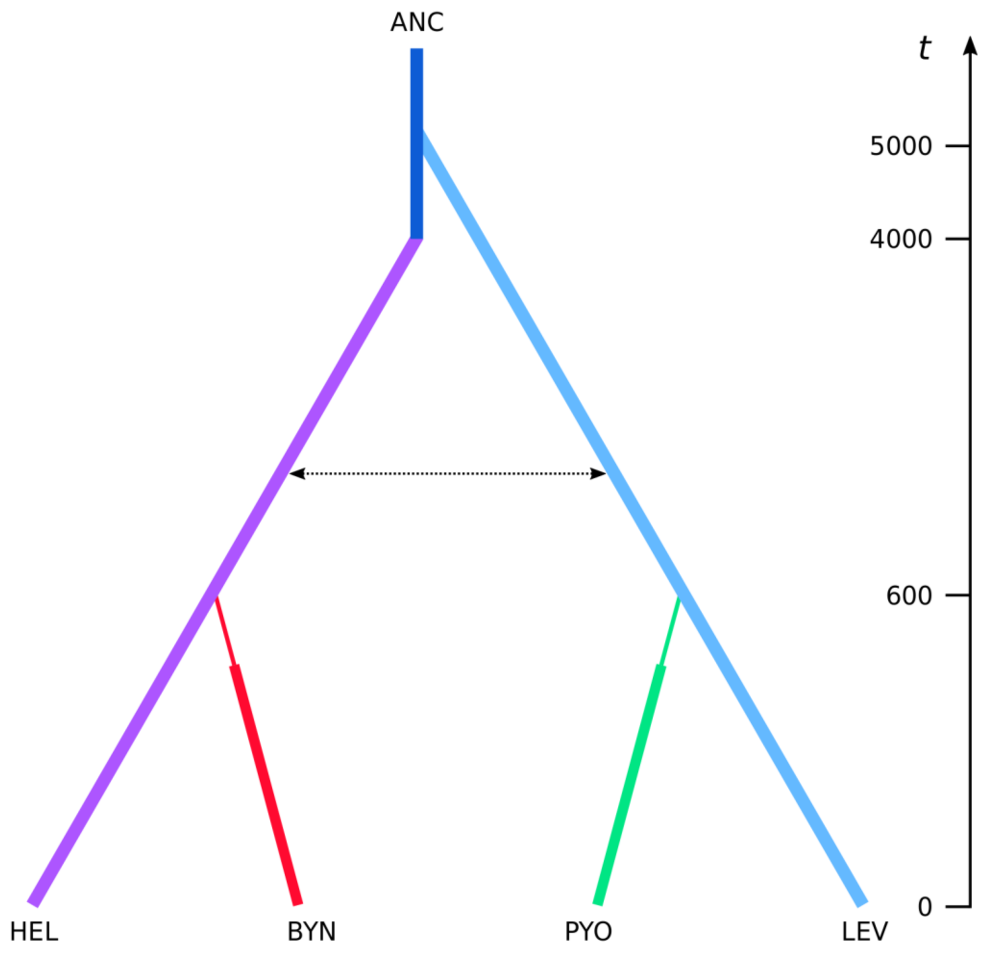

Figure S3. **Schematic representation of the demographic scenario used to simulate *P. pungitius* genomic datasets.** Population codes correspond to the four populations used in this study: Helsinki (HEL), Bynastjärnen (BYN), Pyöreälampi (PYO), White Sea (LEV) and an ancestral population (ANC). Color change indicate a population split at a time *t* (in generations) from present day (0 on the scale). Population size bottlenecks are depicted by a thinning in the given population's branch. Black dashed arrow represents gene flow between HEL and LEV.

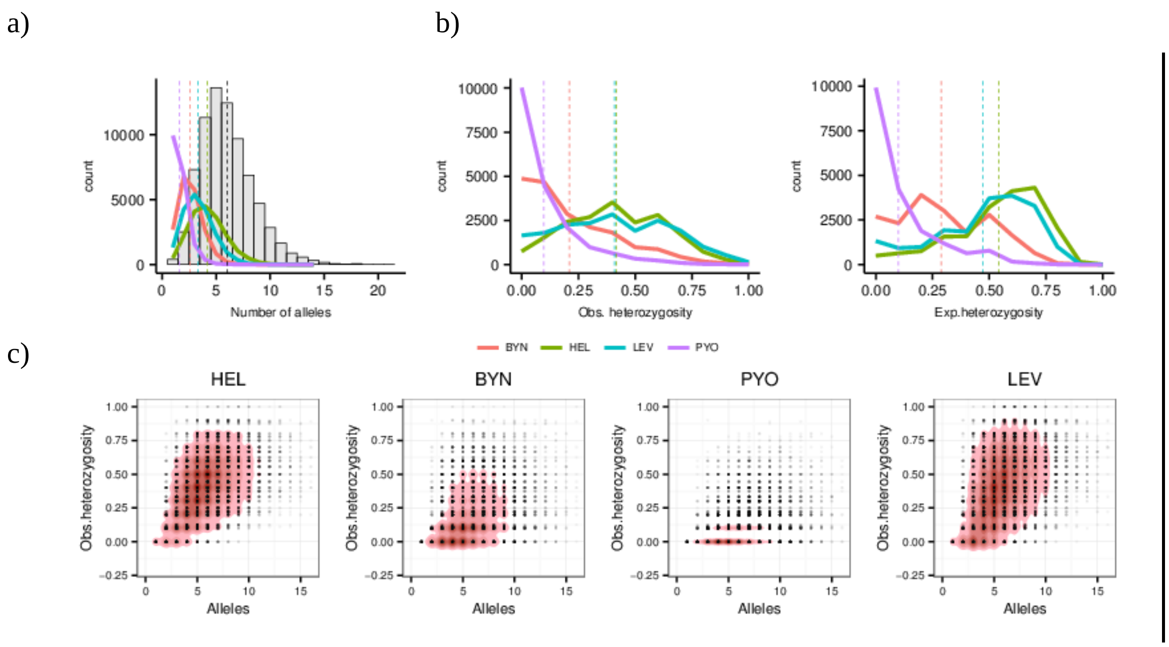

Fig S4. Variation at the microsatellite loci across the four nine-spined stickleback populations. a) Number of alleles per microsatellite locus in individual populations (lines) and in combined data (bars). b) Observed and expected heterozygosity per locus. c) Observed heterozygosity at microsatellite loci (y axis) as a function of total number of alleles (x axis) in the four populations. Values are computed for the 18,824 loci that have data for >80% of samples in each population. Dashed vertical lines indicate the mean value for the corresponding category.

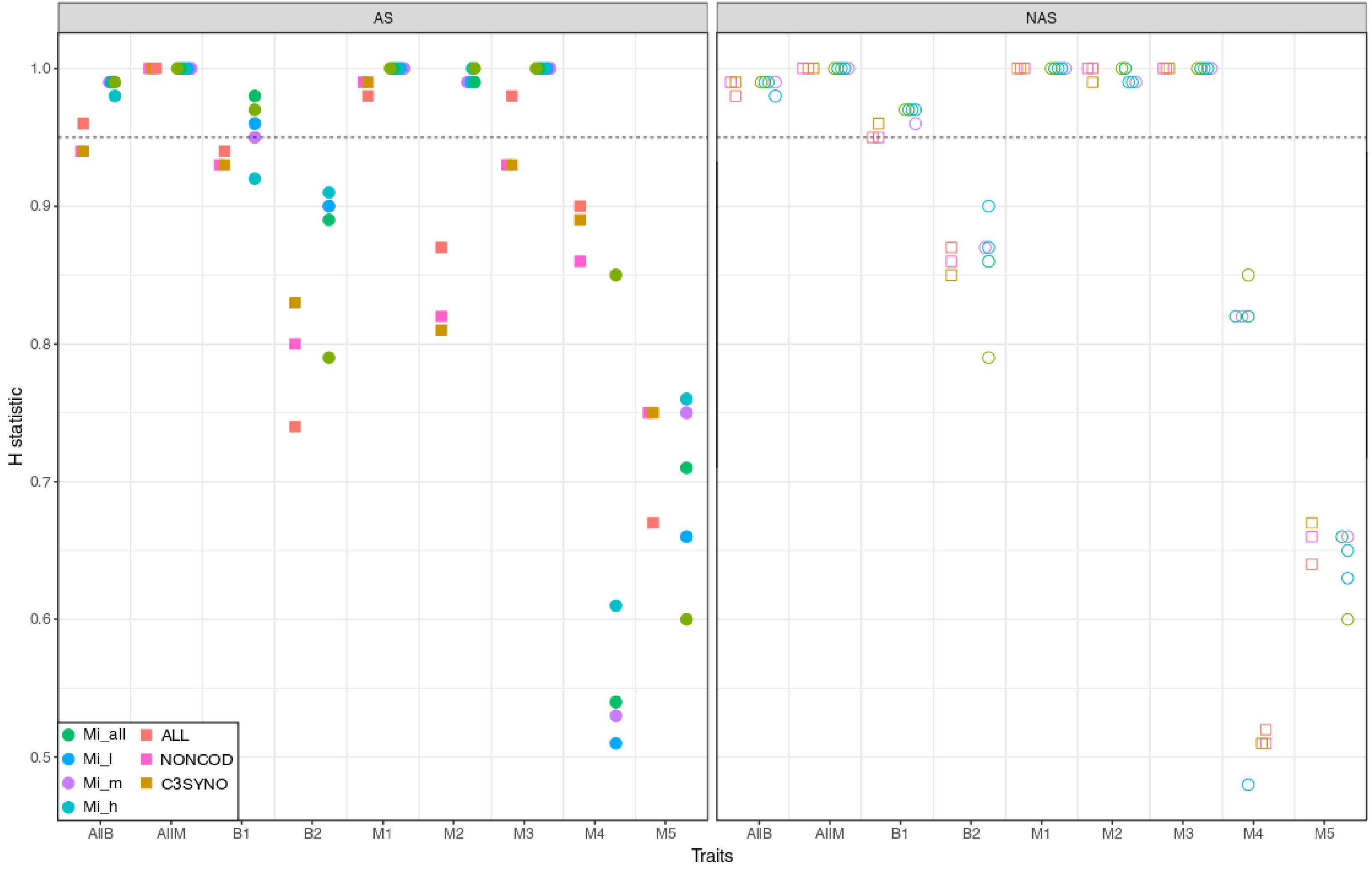

Figure S5. H statistic from Driftsel using ascertained and unascertained markers. Results using ascertained (AS; left panel) and unascertained markers (NAS; right panel) are shown for the following datasets: all *in silico* microsatellites (Mi_all; green circles); *in silico* microsatellites with low (Mi_l; blue circles), intermediate (Mi_m; purple circle) and high (Mi_h; cyan circle) number of alleles ; and for the three datasets with all (ALL; red squares), non-genic (NONCOD; pink squares) and genic (C3SYNO; brown squares) SNPs

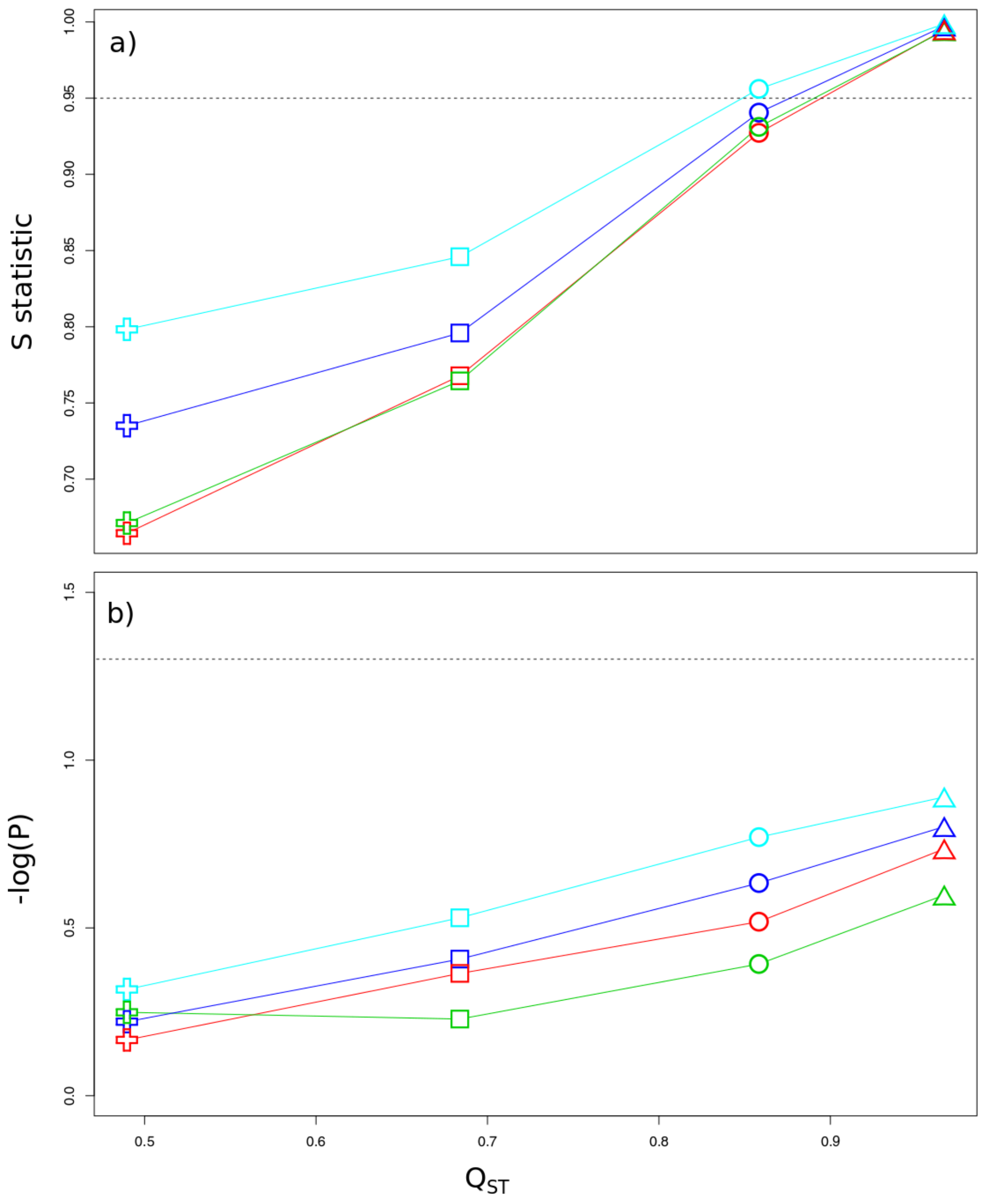

Figure S6. Results of Driftsel and QstFstComp analyses based on simulated data. Averaged results from Driftsel (a) and QstFstComp (b) over 10 simulations replicates are shown for each of the four scenarios and type of marker. Shapes represent the four studied scenarios: i) neutrality ($Q_{ST} = F_{ST}$; open crosses), ii) weak selection ($Q_{ST} > F_{ST}$; open squares), iii) moderate selection ($Q_{ST} > F_{ST}$; open circles) and strong selection ($Q_{ST} >> F_{ST}$; open triangles). Corresponding Q_{ST} values are shown on the x axis. Marker types are color-coded for SNP (green), Mi_l (red), Mi_m (blue) and Mi_h (cyan). The black dashed horizontal lines represent the significant thresholds over which signal of divergent selection is detected using: the S statistic with Driftsel and the –log(P) value for QstFstComp.

	ALL		NONCOD		C3SYNO		Karhunen et al. (2014)	
Trait	S	H	S	H	S	H	S	H
M1	**1**	**1**	**1**	**1**	**1**	**1**	**1**	**1**
M2	**0.97**	**1**	**0.97**	**1**	**0.98**	**0.99**	**0.98**	**1**
M3	**1**	**1**	**1**	**1**	**1**	**1**	**1**	**1**
M4	0.62	0.52	0.60	0.51	0.61	0.51	0.85	0.85
M5	0.79	0.64	0.80	0.66	0.80	0.67	0.73	0.60
All M traits	**1**	**1**	**1**	**1**	**1**	**1**	**1**	**1**
B1	0.91	**0.95**	0.91	**0.95**	0.92	**0.96**	0.91	**0.97**
B2	0.73	0.87	0.73	0.86	0.74	0.85	0.71	0.79
All B traits	0.93	**0.98**	0.92	**0.99**	0.93	**0.99**	0.93	**0.99**
FST (95% CI)	0.52 (0.51;0.53)		0.52 (0.51;0.53)		0.51 (0.50;0.52)		0.35 (0.31;0.38)	

Table S1. S and H statistics from driftsel analysis using three different SNP datasets and unascertained markers.
S and H statistics were computed from the program driftsel [7] based on 2 000 unlinked SNPs in three different SNP-datasets: all (ALL), non-genic (NONCOD) and genic (C3SYNO) SNPs. The same estimates using 12 unlinked microsatellite markers as in Karhunen et al. [8] is provided for comparison. Mean F_{ST} for each dataset is reported at the bottom of the table. Significant values for S and H (i.e. providing signal of divergent selection) are shown in bold.

	Whole dataset		2-4 alleles		5-8 alleles		9-21 alleles	
Trait	S	H	S	H	S	H	S	H
M1	**1**	**1**	**1**	**1**	**1**	**1**	**1**	**1**
M2	**0.98**	**1**	**0.97**	**0.99**	**0.98**	**0.99**	**0.98**	**0.99**
M3	**1**	**1**	**1**	**1**	**1**	**1**	**1**	**1**
M4	0.64	0.82	0.58	0.48	0.64	0.82	0.54	0.82
M5	0.82	0.66	0.79	0.63	0.82	0.66	0.82	0.65
All M traits	**1**	**1**	**1**	**1**	**1**	**1**	**1**	**1**
B1	0.91	**0.97**	0.90	**0.97**	0.91	**0.96**	0.91	**0.97**
B2	0.76	0.86	0.75	0.87	0.76	0.87	0.77	0.90
All B traits	0.94	**0.99**	0.93	**0.98**	0.94	**0.99**	0.94	**0.99**
FST (95% CI)	0.37 (0.36;0.38)		0.43 (0.39;0.46)		0.39 (0.38; 0.40)		0.36 (0.35;0.37)	

Table S2. S and H statistics from driftsel analysis using *in-silico* genotyped microsatellite markers and unascertained markers. S and H statistics were computed from the program driftsel [7] based on the whole microsatellite dataset ("Whole dataset"), or using microsatellites with low ("2-4 alleles"), moderate ("5-8 alleles") or high ("9-21 alleles") number of alleles. Mean F_{ST} for each dataset is reported at the bottom of the table. Significant values for S and H (i.e. providing signal of divergent selection) are shown in bold.

	ALL		NONCOD		C3SYNO	
Trait	F_{ST}-Q_{ST}	*P*	F_{ST}-Q_{ST}	*P*	F_{ST}-Q_{ST}	*P*
M1	0.33	0.186	0.34	0.158	0.34	0.148
M2	0.34	0.158	0.35	0.114	0.35	0.122
M3	0.43	0.100	0.44	0.108	0.45	0.104
M4	-0.12	0.758	-0.12	0.830	-0.11	0.806
M5	0.05	0.836	0.06	0.776	0.06	0.758
B1	0.29	0.288	0.30	0.278	0.30	0.264
B2	0.47	0.076	0.47	0.082	0.47	0.080
FST (95% CI)	0.51 (0.50;0.52)		0.50 (0.49;0.51)		0.50 (0.49;0.51)	

Table S3. F_{ST}-Q_{ST} differences and associated *p*-values from QstFstComp analysis using three different SNP datasets. F_{ST}-Q_{ST} differences and associated *p*-values were computed from the program QstFstComp [55] based on 2 000 unlinked SNPs in three different SNP-datasets: all (ALL), non-genic (NONCOD) and genic (C3SYNO) SNPs. Mean F_{ST} for each dataset is reported at the bottom of the table.

	2-4 alleles		5-8 alleles	
Trait	F_{ST}-Q_{ST}	*P*	F_{ST}-Q_{ST}	*P*
M1	0.37	0.130	0.40	0.124
M2	0.39	0.108	0.42	0.082
M3	0.48	0.096	0.51	0.122
M4	-0.08	0.890	-0.05	0.986
M5	0.09	0.712	0.12	0.598
B1	0.33	0.272	0.36	0.212
B2	0.51	0.088	0.54	0.080
F_{ST} (95% CI)	0.47 (0.42, 0.51)		0.43 (0.42, 0.44)	

Table S4. Results from QstFstComp analysis based on microsatellite markers. The F_{ST}-Q_{ST} differences and associated p-values as estimated using unlinked *in-silico* genotyped microsatellite markers varying in their allele number. Due to the limitation of the software, the analyses are restricted to the loci with low number of alleles. All datasets contained 2000 unlinked microsatellite loci. The baseline F_{ST}-estimates are given at the bottom of the table.

	ALL		NONCOD		C3SYNO		Karhunen et al. (2014)	
Trait	S	H	S	H	S	H	S	H
M1	**1**	**0.98**	**1**	**0.99**	**1**	**0.99**	**1**	**1**
M2	**1**	0.87	**0.98**	0.82	**0.98**	0.81	**0.98**	**1**
M3	**1**	**0.98**	**0.99**	0.93	**0.99**	0.93	**1**	**1**
M4	0.82	0.90	0.84	0.86	0.82	0.89	0.85	0.85
M5	0.76	0.67	0.88	0.75	0.88	0.75	0.73	0.60
All M traits	**1**	**1**	**1**	**1**	**1**	**1**	**1**	**1**
B1	0.91	0.94	0.90	0.93	0.90	0.93	0.91	**0.97**
B2	0.75	0.74	0.75	0.80	0.75	0.83	0.71	0.79
All B traits	0.91	**0.96**	0.89	0.94	0.89	0.94	0.93	**0.99**
FST (95% CI)	0.39 (0.38, 0.40)		0.54 (0.54, 0.55)		0.55 (0.54, 0.56)		0.35 (0.31;0.38)	

Table S5. S and H statistics from Driftsel analysis using three different SNP datasets and ascertained markers. S and H statistics were computed from the program driftsel [7] based on 2 000 unlinked SNPs in three different SNP-datasets: all (ALL), non-genic (NONCOD) and genic (C3SYNO) SNPs. The same estimates using 12 unlinked microsatellite markers as in Karhunen et al. [8] is provided for comparison. Mean F_{ST} for each dataset is reported at the bottom of the table. Significant values for S and H (i.e. providing signal of divergent selection) are shown in bold.

	Whole dataset		2-4 alleles		5-8 alleles		9-21 alleles	
Trait	S	H	S	H	S	H	S	H
M1	**1**	**1**	**1**	**1**	**1**	**1**	**1**	**1**
M2	**0.99**	**0.99**	**0.98**	**0.99**	**0.99**	**0.99**	**1**	**1**
M3	**1**	**1**	**0.99**	**1**	**1**	**1**	**1**	**1**
M4	0.61	0.54	0.57	0.51	0.62	0.53	0.69	0.61
M5	0.81	0.71	0.81	0.66	0.80	0.75	0.81	0.76
All M traits	**1**	**1**	**1.00**	**1.00**	**1**	**1**	**1**	**1**
B1	0.92	**0.98**	0.91	**0.96**	0.91	**0.95**	0.91	0.92
B2	0.81	0.89	0.79	0.90	0.81	0.90	0.81	0.91
All B traits	**0.95**	**0.99**	0.93	**0.99**	0.94	**0.99**	**0.96**	**0.98**
FST (95% CI)	0.29 (0.28,0.30)		0.34 (0.33;0.35)		0.26 (0.25;0.27)		0.17 (0.16;0.18)	

Table S6. S and H statistics from driftsel analysis using *in-silico* genotyped microsatellite markers and ascertained markers. S and H statistics were computed from the program driftsel [7] based on the whole microsatellite dataset ("Whole dataset"), or using microsatellites with low ("2-4 alleles"), moderate ("5-8 alleles") or high ("9-21 alleles") number of alleles. Mean F_{ST} for each dataset is reported at the bottom of the table. Significant values for S and H (i.e. providing signal of divergent selection) are shown in bold.

	ALL		NONCOD		C3SYNO	
Trait	F_{ST}-Q_{ST}	*P*	F_{ST}-Q_{ST}	*P*	F_{ST}-Q_{ST}	*P*
M1	0.33	0.174	0.36	0.152	0.36	0.158
M2	0.37	0.138	0.38	0.112	0.37	0.098
M3	0.46	0.100	0.47	0.118	0.46	0.106
M4	-0.10	0.792	-0.09	0.886	-0.10	0.828
M5	0.07	0.750	0.08	0.710	0.08	0.690
B1	0.31	0.244	0.32	0.254	0.32	0.26
B2	0.49	0.102	0.50	0.070	0.49	0.060
FST (95% CI)	0.49 (0.48;0.49)		0.47 (0.49;0.51)		0.48 (0.47;0.49)	

Table S7. F_{ST}-Q_{ST} differences and associated *p*-values from QstFstComp analysis using three different SNP datasets after deleting ascertained markers.

Population	Genotype at Locus1	Genotype at Locus2
HEL	AB<br>BB<br>AB	AA<br>AB<br>BB
LEV	BB<br>AB<br>BB	AB<br>BB<br>BB
LD = 0 between the two loci in marine populations		
BYN	BB<br>BB<br>BB	BB<br>BB<br>BB
PYO	AA<br>AA<br>AA	AA<br>AA<br>AA
LD = 1 between the two loci in pond populations		
F_{ST}	0.62	0.6

Table S8. Toy illustration of the differences in LD structures in marine and pond populations. The two loci are totally unlinked in marine populations (LEV & HEL). However, they appear to be perfectly linked in the pond populations (BYN & PYO) because of genetic drift (i.e. alleles are fixed in both pond populations). The LD between two loci in the pooled data is also high (0.72), so one of the loci will be pruned out using the sliding window **a**pproach if applied to the pooled data, and a decrease the mean F_{ST} will ensue.

		Driftsel	QstFstComp
Scenario (i): neutral pattern	SNP	0	0
	Mi_l	0	0
	Mi_m	0	0
	Mi_h	1	0
Scenario (ii): weak selection	SNP	1	2
	Mi_l	1	1
	Mi_m	1	2
	Mi_h	2	3
Scenario (iii): moderate selection	SNP	5	1
	Mi_l	5	0
	Mi_m	6	1
	Mi_h	8	1
Scenario (iv): strong selection	SNP	10	1
	Mi_l	10	0
	Mi_m	10	1
	Mi_h	10	1

Table S9. **Number of detected signals of selection among 10 simulation replicates using Driftsel and QstFstComp.** For the neutral scenario (i) the reported quantity is a measure of false positives. For scenarios (ii)-(iv) with selection, the reported quantity is a measure of true positives.
